# Application of Response Surface Methodology to Study the Effects of Brisket Fat, Soy Protein Isolate, and Cornstarch on Nutritional and Textural Properties of Rabbit Sausages

**DOI:** 10.1155/2017/7670282

**Published:** 2017-06-19

**Authors:** Joseph M. Wambui, Edward G. Karuri, Margaret M. M. Wanyoike

**Affiliations:** ^1^Department of Food Science, Nutrition and Technology, University of Nairobi, Nairobi 29053-00625, Kenya; ^2^Department of Animal Production, University of Nairobi, Nairobi 29053-00625, Kenya

## Abstract

The effects of brisket fat, soy protein isolate, and cornstarch on chemical and textural properties of rabbit sausages were studied using surface response methodology. Sausage samples were prepared using a five-level three-variable Central Composite Rotatable Design with 16 combinations, including two replicates of the center point, carried out in random order. The level of brisket fat (BF), soy protein isolate (SPI), and cornstarch (CS) in the sausage formulation ranged within 8.3–16.7%, 0.7–2.3%, and 1.3–4.7%, respectively. Increasing BF decreased moisture and ash contents but increased protein and fat contents of the sausages (*p* < 0.05). Increasing SPI increased moisture content but decreased ash and carbohydrate contents of the sausages (*p* < 0.05). Increasing CS increased carbohydrate content (*p* < 0.05). Increasing BF increased hardness, adhesiveness, cohesiveness, and chewiness but decreased springiness (*p* < 0.05). SPI addition increased springiness but decreased adhesiveness, cohesiveness, and chewiness (*p* < 0.05). In conclusion, varying the levels of BF and SPI had a more significant effect on chemical and textural properties of rabbit sausages than CS.

## 1. Introduction

Recently, meat has been subject to a lot of negative publicity. This has been attributed to its contents, mainly fat, saturated fatty acids, and cholesterol, and their association with chronic diseases, such as cardiovascular diseases, some types of cancer, and obesity [1]. This has led consumers to demand more health oriented functional meat products that are low in these components [2]. In response to these demands, the meat industry has in recent years endeavored to develop healthier meat products that incorporate health enhancing ingredients such as carotenoids and unsaturated fatty acids [3, 4]. Much attention has been paid to the development of habitually consumed products with physiological functions that promote human health and reduce the prevalence of chronic diseases, such as cardiovascular diseases [5].

Because of the recent advances, there has been a shift from traditional sources of meat to newer sources such as fish, poultry, and rabbit whose meat is deemed healthier. Among these sources, rabbit meat is often recommended because it fits well with the current consumer demand for a low-fat meat with high unsaturated fatty acid, phosphorus, and iron contents while the sodium levels are low [6, 7]. It is also characterized by its lower energetic value and cholesterol compared with beef and poultry [6, 8]. In addition, rabbit meat consumption has been proposed as one of the means by which consumers can acquire bioactive compounds. The content of n-3 polyunsaturated fatty acids (PUFA), conjugated linoleic acid (CLA), and vitamins in rabbit meat can be easily increased by modifying the diet of the rabbits [7, 9, 10]. Both selenium and iron are also responsive to dietary supplementation in rabbits [11].

According to FAOSTAT, more than 1.6 and 1.1 billion rabbits were produced and slaughtered for meat, respectively, in 2014 [12]. This is compared to 1.1 and 0.8 billion rabbits which were produced and slaughtered, respectively, in 2004 [12]. This translates to 45.4% and 37.5% increase in rabbits produced and slaughtered for meat, respectively. Evidently, production of rabbit has risen in the last decade, which translates to increased consumption for rabbit meat globally. Given that rabbits reproduce rapidly, farmers have an advantage that they can capitalize on to satisfy such demands [13]. In turn, this enhances sustainable rabbit meat production. Although the increased demand is evident, data on rabbit meat consumption are scarce. Available data show that consumption ranges from 0.93 to 4.4 kg/person in Europe, where rabbit meat is mostly consumed [14].

Despite the nutritional health benefits, current demand and production of rabbit meat continues to be low, especially when compared to other meats, such as chicken, whose demand was 100 billion in 2014 [12]. The low demand can be attributed to the fact that rabbit production has remained cottage industry where only a few rabbits are produced and the rabbit meat is continually dominated by small scale farmers who maintain a maximum of 50 breeding rabbits [15]. Only a few meat processors have focused on introducing processed rabbit meat products for the consumers [16]. Very low quantities of rabbit meat are in fact marketed in form of processed products (i.e., ready-to-cook, ready-to-eat meals, etc.) unlike whole carcass or at least as cut-up parts [17]. The processed rabbit meat products (e.g., meat patties and sausages) available currently are made from coarsely ground meat, which have not gained much interest in the marketplace [17]. A recent study on commercial rabbit sausages in Kenya found out that they are of low quality [18]. Therefore, even though there is a big demand for meat of nutritional health benefits, rabbit meat has not been able to appeal to most consumers. This is a big challenge for the rabbit meat processors that needs to be addressed.

Strategies to increase the demand of rabbit meat include diversification of rabbit meat products and an understanding of the contribution of the meat to these products [17, 19]. Value addition to the rabbit meat products not only would provide the much needed nutritional components, but can increase consumer convenience through decreasing preparation time and minimizing preparation steps [20, 21]. The popularity of convenience foods among modern consumers may provide an answer to a long-standing question of how to increase the demand for rabbit meat. One of the most popular meat products is the sausage, but to make them more appealing to the modern consumer, an optimal rabbit sausage formulation has been recommended [18].

Development of an optimal formulation requires that the effects of ingredients in the formulation are known at first. This will then allow for mathematical modelling of the optimal formulation. Given that the issues underlying the marketing strategies of rabbit meat include the increasing importance of quality and sensory properties of food in general [22], then effects of the added ingredients on properties such as chemical and texture should be studied. Furthermore, success of processed meat products depends majorly on appropriate quality raw materials, correct formulation, and optimum processing [21].

Several studies have examined the use of various functional ingredients or adjuncts, such as soy protein isolate, cornstarch, and beef fat in sausage formulations. Soy protein isolate is commonly used as a binder to reduce processing cost and water loss, to increase yield and viscosity, and to stabilize the emulsion of emulsion-type meat products [3]. In addition to technological properties, soy protein has numerous nutritional benefits which have been extensively reviewed [23]. Cornstarch has been studied as a fat replacer in meat products [24, 25]. On the other hand, fat acts as a reservoir for flavour compounds and contributes to product texture [26]. Beef fat is one of the animal fats that are used in meat products. It contains 3 *µ*g of CLA per gram of fat [27]. CLA has numerous health benefits that have been extensively reviewed [28, 29]. CLA are predominant in ruminant meat and meat products [30] and can also be increased in foods by heating, such as cooking and processing [31]. Since sausages are heated before cooking, use of beef fat in sausage processing can be a good source of CLA.

Response surface methodology (RSM), a powerful mathematical and statistical technique for testing multiple process variables and their interactive and quadratic effects, is useful in solving multivariable equations obtained from experiments simultaneously [32]. In the analysis of interactions between the responses (dependent variables) and the factors (independent variables) of experiment, this technique provides an advantage of the reduction in the number of experiments as compared to the full experimental design [32]. RSM has been used for the simultaneous analysis of the effects of added ingredients on the physiochemical properties of sausages [4, 33–35]. These studies show that RSM can help in predicting the combined effects of ingredients on the properties. Nevertheless, this technique has not been applied in processed rabbit meat products. Therefore, the objective of the present study was to assess the effects of brisket fat, soy protein isolate, and cornstarch on chemical and textural properties of rabbit sausages by applying the surface response methodology.

## 2. Material and Methods

### 2.1. Raw Materials

Rabbit meat from different parts of rabbit carcass was obtained from three-month-old California White bucks donated by the University of Nairobi, Department of Animal Production. Brisket fat (BF) was purchased from Dagoretti Slaughterhouse, Nairobi, Kenya. Cornstarch (CS) (Pradip Enterprises E.A. Ltd., Nairobi, Kenya), soy protein isolate (SPI) (Pulsin Ltd., Gloucester, United Kingdom), spices (Deepa Industries Ltd., Nairobi, Kenya), and other additives were purchased from local retail outlets.

### 2.2. Sample Preparation

Sausage samples were prepared based on a five-level three-variable Central Composite Rotatable Design (CCRD) with 16 combinations, including two replicates of the center point, carried out in random order. This experimental design was generated using Design Expert version 9 (Stat-Ease Inc., Minnesota, USA). The combinations were prepared by varying levels of BF, SPI, and CS (Table 1). The rabbit meat and BF were chilled overnight in separate polyethylene bags at 4°C. The chilled lean meat was ground through a 5 mm plate and then a 3 mm plate. The BF was diced into pieces of 10–20 mm and then ground through a 3 mm plate. For each combination, the two were mixed together depending on the levels in Table 1 and chopped at medium speed. Ice water at five percent was added and then chopping continued for four minutes. The target moisture content of the product was 63%, which is the average content in frankfurter sausages [36]. CS and SPI were then added at percentages shown in Table 1. The remaining five percent ice water, seasonings, and spices were also added at this stage. Seasonings and spices included sodium chloride (2.27%), coriander (2%), white pepper (2%), ginger (0.3%) garlic (0.5%), monosodium glutamate (1.5%), sodium nitrite (0.3%), sodium tri-poly-phosphate (0.5%), and ascorbic acid (0.05%). Chopping was continued until the final temperature of the batter reached 12°C.

### 2.3. Sample Preparation for Analysis

The sausage batter was manually stuffed into 21 mm collagen casings. Sausages were hand-linked at 10 cm intervals and allowed to dry at room temperature for 2 h, which is a common practice in sausage processing [37]. The drying was carried out in a hygienic environment to prevent contamination. After drying, the samples were vacuum-packed and stored in a cooler at 4°C until further analysis. Approximately 20 sausages were obtained for each combination. For analysis, nine out of the 20 sausages were randomly sampled. The nine sausages were further randomly divided into three equal groups. Each group was subjected to either chemical or textural analysis. Before analysis, the sausages were heated in boiling water for five minutes [38].

### 2.4. Chemical Analysis

The chemical composition of the samples was determined by proximate analysis according to official methods [39]. The three samples were ground together and the homogenate was used for analysis. Crude protein and crude lipid contents were measured by Kjeldahl and Soxhlet methods, respectively. Ash content was determined by ashing the samples overnight at 550°C. Moisture content was determined by drying the samples overnight at 105°C and carbohydrate content was calculated by computing the difference.

### 2.5. Textural Analysis

Textural properties were evaluated using TA.XT* plus *Texture Analyzer (Stable Micro Systems, UK). Each of the three sausages was divided into central cores of 1 cm height and 1.3 cm diameter. To improve the ease of core preparation, the analysis was performed at a uniform temperature of 20-21°C [40]. Three well-shaped cores were sampled and compressed to 50% of their original height two times using a 75 mm compression platen and 50 kgf load cell. The compression parameters included a constant speed of 3.0 mm/s, test speed of 1.0 mm/s, posttest speed of 3.0 mm/s, and prefixed strain of 75%. The texture profile tests were hardness (maximum force required to compress the sample), adhesiveness (the work necessary to overcome the attractive forces between the surface of a food and surface of other materials which it comes in contact with), springiness (ability of the sample to recover its original form after the deforming force was removed), cohesiveness (extent to which the sample could be deformed prior to rupture), and chewiness (work necessary to masticate the sample for swallowing) [41].

### 2.6. Statistical Analysis

Data were analyzed using Design Expert version 9 (Stat-Ease Inc., Minnesota, USA). A 3-factor 5-level Central Composite Rotatable Experimental Design [42] with two center points was used to develop predictive models for chemical and textural score parameters of rabbit sausages. The three factors (processing variables), levels, and experimental design in terms of coded and uncoded are those presented in Table 1. The following second-order polynomial equation of function *X*_*i*_ was fitted for each factor assessed where *Y* was the estimated response, *β*_0_, *β*_*i*_, *β*_*ii*_, and *β*_*ij*_ were constant coefficients, *k* was the number of factor variables, and *x*_*i*_, *x*_*ii*_, and *x*_*ij*_ represented the linear and interactive effects of the independent variables, BF, SPI, and CS, respectively.

(1) $$\begin{array}{c} Y=\beta_{0}+\overset{k}{\underset{i=1}{\sum }}\beta_{i}x_{i}+\overset{k}{\underset{ii=1}{\sum }}\beta_{ii}x_{ii}^{2}+\overset{k}{\underset{i=1}{\sum }}\;\overset{k}{\underset{j=1}{\sum }}\beta_{ij}x_{i}x_{j}. \end{array}$$

The analysis was performed using uncoded units. For each factor assessed, the variance was partitioned into linear, quadratic, and interaction terms in order to assess the fit of the second-order polynomial function and the relative significance of these terms. The significance of the equation parameters for each response variable was assessed by analysis of variance. Regression analysis and nonsignificant lack of fit were also determined. Several response surfaces in form of 3-dimensional representations were drawn to show the effect of two given independent variables on a given response, by imposing a constant value equal to mid-level of the third variable. The effects of the variables BF, SPI, and CS content were classified as first-order (linear), second-order (quadratic), and interactive.

## 3. Results and Discussion

### 3.1. Effects on Chemical Properties

**Figure 1:**
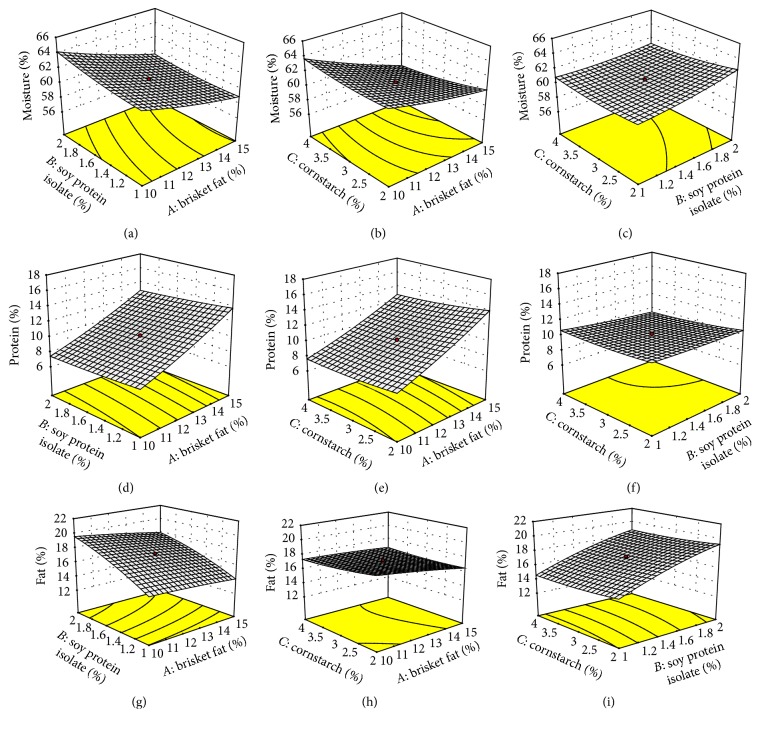
Effect of (a) brisket fat and soy protein isolate, (b) brisket fat and cornstarch, and (c) soy protein isolate and cornstarch on moisture content, (d) brisket fat and soy protein isolate, (e) brisket fat and cornstarch, and (f) soy protein isolate and cornstarch on protein content, and (g) brisket fat and soy protein isolate, (h) brisket fat and cornstarch, and (i) soy protein isolate and cornstarch on fat content along with the second-order polynomial model equations predicting effects of the variables. ((a), (b), (c)) Moisture = 60.77 − 2.01*A* + 0.59*B* − 0.28*C* − 0.29*AB* − 0.66*AC* − 0.39*BC* + 0.24*A*^2^ + 0.28*B*^2^ + 0.32*C*^2^. ((d), (e), (f)) Protein = 10.14 + 2.8*A* − 0.38*B* − 0.39*C* − 0.11*AB* − 0.2*AC* − 0.25*BC* + 0.29*A*^2^ + 0.093*B*^2^ + 0.21*C*^2^. ((g), (h), (i)) Fat = 17.15 + 0.63*A*  + 2.11*B*  − 0.23*C*  − 0.20*AB*  + 0.28*AC*  − 0.024*BC*  − 0.096*A*^2^  − 0.41*B*^2^  + 0.19*C*^2^.

Mean percent moisture, protein, fat, ash, and carbohydrates of rabbit sausage samples and the effects of added brisket fat (BF), soy protein isolate (SPI), and cornstarch (CS) are presented in Table 2. Moisture, protein, fat, ash, and carbohydrate contents ranged from 57.3% to 64.9%, 7.0% to 14.3%, 14.1% to 20.0%, 2.4% to 2.7%, and 3.7% to 13.0%, respectively. The three-dimensional representation of some of the effects on chemical properties is shown in Figures 1(a)–1(i). Increasing BF in the ingredient formulation decreased moisture and ash contents but increased protein and fat contents (*p* < 0.05). Similar results have been reported where increasing beef fat from 5% to 20% significantly reduced moisture content of beef frankfurter sausages [43]. In the present study, BF was increased from 8.3% to 16.7%. The effect of BF on moisture content can be attributed to an inverse relationship between fat and moisture contents in this case. Such a relationship has been reported between beef tallow and moisture content in cooked beef balls, in which case fat level in the formulation ranged from 0 to 19% [44].

Although the present results showed a significant effect of addition of BF on the protein content of the sausages, there is a difference with some previous reports in literature. In one such case where cooked beef patties were studied, increasing fat content from 10 to 30% decreased protein content [45]. Normally, when the meat content is kept constant, changes in protein content of meat products can be attributed to addition of ingredients [4]. In the present study, the meat content depended on the summed percentage of BF, SPI, and CS. In addition, the highest fat content in the present study was nearly 12% less than that used in the beef patties [45]. Therefore, variations in ingredient formulation of the rabbit sausages and differences in the amount of fat used compared to other studies may have led to the observed differences. The effect of BF was as expected and corresponded with previous studies that reported that increasing fat levels in a formulation increases the fat content of the end product [45]. This may be expected because rabbit meat has relatively low-fat content [46] while SPI and CS have less than 1% fat content [47, 48].

Increasing SPI from 0.7% to 2.3% significantly increased moisture content but decreased ash and carbohydrate contents of rabbit sausages (*p* < 0.05). However, fat and protein contents were not affected by SPI level (*p* > 0.05). The present results are similar to those of a previous study where increasing SPI to 2% increased moisture content, but not protein and fat content of pork sausages [49]. The increase in moisture content is attributed to good gelling properties of SPI. The lack of effect of SPI on fat is similar to previous results in which it was found that soy protein at 4% levels did not affect the fat content in cooked beef sausages [50]. The present results may be attributed to the levels of SPI relative to those of BF in the formulation. The lower levels of SPI than BF may not have been sufficient to substitute the fat in the final product. Hence there is a lack of any effect of SPI on the fat content of the sausages. However, there are still some differences with other studies. In one such study, increase of SPI to 2% in bologna type sausages did not result in differences in protein, moisture, and ash content, although fat content decreased [51]. In another study, frankfurter type sausage with 2% SPI had lower fat and moisture contents and higher protein content than in the controls [52]. On the other hand, low-fat pork sausages with 1.5% SPI had similar contents of fat, moisture, and protein with the control [53].

Although soy protein products are used to extend or replace animal proteins [54], the results from this study may indicate that, at levels of about 2%, SPI does not serve this function in rabbit sausages. In addition, this level is not enough to act as a fat replacer. However, increased moisture at this level confirms that indeed SPI is a good gelling agent. Soy proteins are hydrophilic (absorb and retain water) and can therefore form a gel that act as a matrix for holding moisture [55]. It has been found that SPI can improve water holding capacity during cooking processes [3]. The only effect observed from the addition of CS was an increase in carbohydrate content (*p* < 0.05). This may be expected because the carbohydrate content of SPI and CS is about 8% and 86%, respectively, and meat contains low amounts of carbohydrates [36, 56, 57]. The high content of carbohydrates in the CS therefore contributed to the increase in carbohydrate content of the sausages.

### 3.2. Effects on Textural Properties

**Figure 2:**
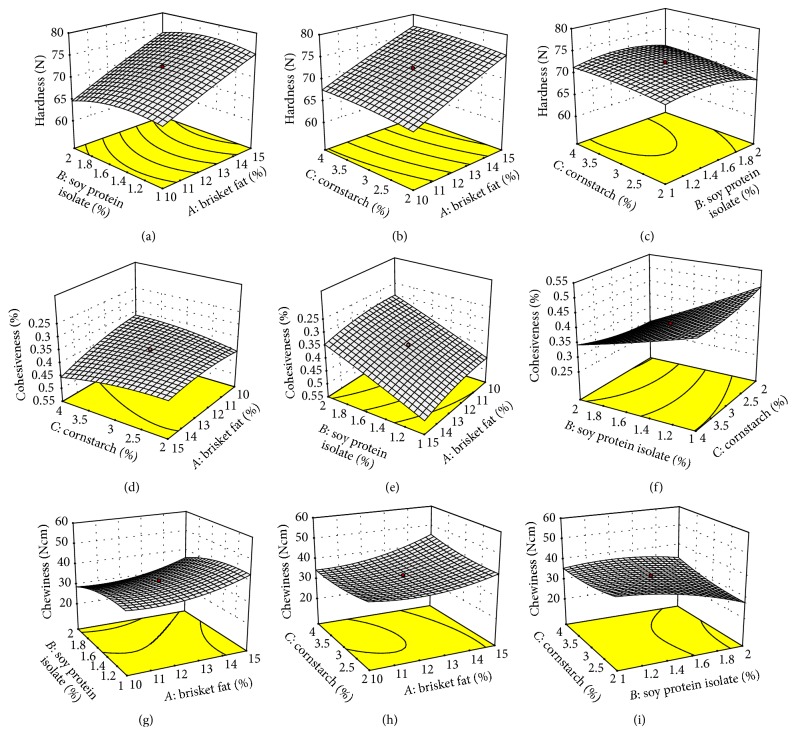
Effect of (a) brisket fat and soy protein isolate, (b) brisket fat and cornstarch, and (c) soy protein isolate and cornstarch on hardness, (d) brisket fat and cornstarch, (e) brisket fat and soy protein isolate, and (f) soy protein isolate and cornstarch on chewiness, and (g) brisket fat and soy protein isolate, (h) brisket fat and cornstarch, and (i) soy protein isolate and cornstarch on cohesiveness along with the second-order polynomial model equations predicting effects of the variables. ((a), (b), (c)) Hardness = 72.22 + 64.79*A* − 0.53*B* + 0.85*C* + 0.44*AB* − 0.04*AC* + 0.27*BC* − 0.22*A*^2^ − 1.51*B*^2^ − 0.44*C*^2^. ((d), (e), (f)) Cohesiveness = 0.38 + 0.023*A* − 0.076*B* + 1.52 × 10^−3^*C* − 9.89 × 10^−4^*AB* + 0.022*AC* + 0.023*BC* + 0.013*A*^2^ + 3.07 × 10^−3^*B*^2^ + 0.012*C*^2^. ((g), (h), (i)) Chewiness = 35.28 + 3.62*A* − 3.57*B* + 3.40 × 10^−1^*C* + 1.90 × 10^−2^*AB* + 1.83*AC* + 2.45*BC* + 3.07*A*^2^ − 2.38*B*^2^ + 1.36*C*^2^.

The mean of the studied texture profiles of rabbit sausage samples and the effects of added BF, SPI, and CS are shown in Table 3. Hardness, adhesiveness, springiness, cohesiveness, and chewiness ranged within 61.3–78.3 N, −0.9–−0.2 Ns, 1.0–1.6 mm, 0.3–0.5, and 23.9–51.6 Nmm, respectively. The three-dimensional representation of some of the effects on chemical properties is shown in Figures 2(a)–2(i). Addition of brisket fat increased hardness, adhesiveness, cohesiveness, and chewiness but decreased springiness (*p* < 0.05). Brisket fat contains large fat globules which translates to less surface area or volume being covered by proteins thus making bonding in the sausage matrix less likely and hence little resistance [58]. The result is a soft product. However, the present results showed an increased hardness, which could indicate a possibility of increased bonding between rabbit meat proteins and brisket fat making the sausages harder. Furthermore, fat and moisture have an inverse relationship between fat and moisture in meat products [59]. Increasing fat may have resulted in water being substituted resulting in harder sausages. On the other hand, different fats when used to formulate different meat products result in varying textural properties [60]. Nevertheless, the present results are similar to those which showed that levels of fat from beef and values of hardness, adhesiveness, cohesiveness, and chewiness had a direct relationship [44]. The inverse relationship between BF and springiness corresponds to a previous report that increase in fat decreases springiness of sausages [61].

SPI addition increased springiness but decreased adhesiveness, cohesiveness, and chewiness (*p* < 0.05). SPI had no effect on hardness (*p* > 0.05). Unfortunately, there is no consensus from literature about the effect of soy protein on texture of processed meats [62]. Nevertheless, the present results on hardness and cohesiveness seem to differ with some of the identified studies. Using similar measurements of texture profile analysis (TPA), higher values for hardness and cohesiveness of samples with SPI than control have been found [62, 63]. On the other hand, it has been reported that increasing the concentration of soy protein flour from 2 to 5% significantly decreased the hardness of beef patties but did not influence the cohesiveness of the samples, both measured with the use of TPA compression test [64]. On the other hand, it was found that the addition of soy protein decreased the hardness of sausages [65]. These differences may be expected since meat from different species was used. Therefore, the meat system in which soy protein is used may be an important factor in determining the textural changes. Addition of cornstarch had no effect on textural properties (*p* > 0.05).

## 4. Conclusion

In the present study, significant effects of brisket fat, soy protein isolate, and cornstarch were observed. By varying the levels of brisket fat and soy protein isolate within 8.3–16.7% and 0.7–2.3%, respectively, more effects were observed than when cornstarch was varied within 1.3–4.7%. In addition, the effects of brisket fat and soy protein isolate were observed to be opposite to each other. By comparison, the effects of these ingredients in the rabbit sausages and effects reported in studies that carried out similar investigations in products from other animal species similarities were observed. However, differences were also observed, and these differences point to the fact that these effects may result in products that are technologically different given the source of the meat. To further understand the effects of various ingredients in rabbit meat products, other ingredients popularly used in meat processing and even those that are being developed for use should be studied. This will lead to the development of a full spectrum of the effects of the ingredients in rabbit meat products and thus aid rabbit meat processors to compete effectively with other meat processors. This may lead to a positive shift in the demand for rabbit meat.

## Figures and Tables

**Table 1:** Mixture design of brisket fat, soy protein isolate, and cornstarch to evaluate the effects of process variables and experimental responses for nutritional and textural properties of rabbit sausages.

Experimental order		Factor levels (coded)			Factor levels (uncoded)^*∗*^		
Standard order	Run order	BF	SPI	CS	BF (%)	SPI (%)	CS (%)
10	1	1.7	0	0	16.7	1.5	3.0
3	2	−1	1	−1	10.0	2.0	2.0
1	3	−1	−1	−1	10.0	1.0	2.0
11	4	0	−1.68	0	12.5	0.7	3.0
9	5	−1.68	0	0	8.3	1.5	3.0
6	6	1	−1	1	15.0	1.0	4.0
15	7	0	0	0	12.5	1.5	3.0
12	8	0	1.7	0	12.5	2.3	3.0
16	9	0	0	0	12.5	1.5	3.0
8	10	1	1	1	15.0	2.0	4.0
13	11	0	0	−1.68	12.5	1.5	1.3
2	12	1	−1	−1	15.0	1.0	2.0
14	13	0	0	1.7	12.5	1.5	4.7
7	14	−1	1	1	10.0	2.0	4.0
5	15	−1	−1	1	10.0	1.0	4.0
4	16	1	1	−1	15.0	2.0	2.0

^*∗*^Percentage of ingredient in each sausage batter; BF: brisket fat; SPI: soy protein isolate; CS: cornstarch.

**Table 2:** Mean values for the proximate composition of rabbit sausage samples and the significance of the regression models (*F* values) and the effects of the processing values on chemical composition.

Run	Processing variables			Mean proximate compositions of rabbit sausage samples (16 runs)					*F* values and the effect of processing variables						
	BF (%)	SPI (%)	CS (%)	MC (%)	PC (%)	FC (%)	AC (%)	CC (%)	Source of variance	DF	MC (%)	PC (%)	FC (%)	AC (%)	CC (%)
1	10.0	1.0	2.0	62.3	8.0	15.2	2.7	11.9	Model	9	12.54^*∗*^	14.9^*∗*^	12.75^*∗*^	19.8^*∗*^	10.84^*∗*^
2	15.0	1.0	2.0	60.2	13.9	14.1	2.5	9.4	*R* ^2^		0.95	0.89	0.88	0.92	0.86
									Linear						
3	10.0	2.0	2.0	64.9	7.2	19.7	2.6	5.5	*A*	1	91.91^*∗∗*^	127.12^*∗∗*^	8.84^*∗*^	129.9^*∗∗*^	0.04
4	15.0	2.0	2.0	62.2	14.3	17.5	2.4	3.7	*B*	1	8.03^*∗*^	2.32	98.14^*∗∗*^	22.67^*∗*^	70.76^*∗*^
5	10.0	1.0	4.0	63.2	7.2	15.1	2.6	11.9	*C*	1	1.85	2.44	1.21	4.49	11.65^*∗*^
									Cross						
6	15.0	1.0	4.0	58.9	13.9	14.8	2.4	10.0	*AB*	1	1.11	0.11	0.52	1.37	2.70
7	10.0	2.0	4.0	64.7	7.0	19.2	2.5	6.6	*AC*	1	5.79	0.38	1.00	4.98	2.45
8	15.0	2.0	4.0	58.9	11.7	18.5	2.4	8.6	*BC*	1	2.07	0.59	0.01	0.28	3.46
									Quadratic						
9	8.3	1.5	3.0	64.7	7.3	18.3	2.7	7.0	*A* ^2^	1	0.87	0.93	0.14	2.31	1.85
10	16.7	1.5	3.0	57.3	15.6	15.7	2.4	9.0	*B* ^2^	1	1.19	0.09	2.56	4.69	0.00
11	12.5	0.7	3.0	60.6	11.7	12.1	2.6	13.0	*C* ^2^	1	1.61	0.51	0.54	2.41	4.66
12	12.5	2.3	3.0	61.7	10.2	20.0	2.5	5.6							
13	12.5	1.5	1.3	61.3	11.8	19.1	2.4	5.5	Residual	6					
14	12.5	1.5	4.7	61.3	10.7	16.5	2.4	9.1	Lack of fit	5	63.75	20.85	3.94	26.37	142.72
15	12.5	1.5	3.0	60.9	10.2	16.8	2.5	9.6	Pure error	1					
16	12.5	1.5	3.0	60.8	9.9	17.4	2.5	9.4							

BF: brisket fat; SPI: soy protein isolate; CS: cornstarch; MC: moisture content; PC: protein content; FC: fat content; AC: ash content; CC: carbohydrate content; DF: degrees of freedom; A: brisket fat; B: soy protein isolate; C: cornstarch. ^*∗*^*p* < 0.05; ^*∗∗*^*p* < 0.001.

**Table 3:** Mean values for the texture profile of rabbit sausage samples and the significance of the regression models (*F* values) and the effects of the processing values on chemical composition.

Run	Processing variables			Mean texture values of rabbit sausage samples (16 runs)					*F* values and the effect of processing variables						
	BF (%)	SPI (%)	CS (%)	HA (N)	AD (Ns)	SP (mm)	CO	CH (Nmm)	Source of variance	DF	HA (N)	AD (Ns)	SP (mm)	CO	CH (Nmm)
1	10.0	1.0	2.0	65.1	−0.5	1.3	0.5	44.0	Model	9	10.55^*∗*^	14.09^*∗*^	9.88^*∗*^	7.01^*∗*^	7.07^*∗*^
2	15.0	1.0	2.0	73.5	−0.2	1.2	0.5	41.8	*R* ^2^		0.85	0.89	0.84	0.78	0.78
									Linear						
3	10.0	2.0	2.0	61.3	−0.9	1.5	0.3	26.3	*A*	1	84.43^*∗∗*^	30.51^*∗*^	7.72^*∗*^	4.83	16.61^*∗*^
4	15.0	2.0	2.0	74.1	−0.6	1.5	0.3	33.1	*B*	1	1.05	79.18^*∗*^	47.64^*∗*^	51.51^*∗*^	16.15^*∗*^
5	10.0	1.0	4.0	66.7	−0.5	1.2	0.4	33.1	*C*	1	2.68	3.05	0.64	0.02	0.15
									Cross						
6	15.0	1.0	4.0	77.6	−0.3	1.1	0.5	47.0	*AB*	1	0.41	0.87	0.01	0.01	0.01
7	10.0	2.0	4.0	66.6	−0.7	1.5	0.4	34.1	*AC*	1	0.01	1.59	0.01	2.47	2.49
8	15.0	2.0	4.0	76.6	−0.7	1.4	0.4	39.3	*BC*	1	0.15	0.68	0.03	2.72	4.46
									Quadratic						
9	8.3	1.5	3.0	64.5	−0.9	1.6	0.4	36.3	*A* ^2^	1	0.12	0.31	12.57^*∗*^	1.05	8.12^*∗*^
10	16.7	1.5	3.0	78.3	−0.5	1.3	0.5	51.6	*B* ^2^	1	5.67	7.26^*∗*^	6.49^*∗*^	0.06	4.87
11	12.5	0.7	3.0	68.6	−0.2	1.0	0.5	33.2	*C* ^2^	1	0.49	1.05	0.78	0.94	1.59
12	12.5	2.3	3.0	66.9	−0.7	1.3	0.3	23.9							
13	12.5	1.5	1.3	71.3	−0.7	1.3	0.4	40.2	Residual	6					
14	12.5	1.5	4.7	70.2	−0.5	1.4	0.4	38.0	Lack of fit	5	19.66	99.59	52.14	8.99	7.70
15	12.5	1.5	3.0	71.9	−0.6	1.3	0.4	34.4	Pure error	1					
16	12.5	1.5	3.0	72.6	−0.6	1.3	0.4	36.2							

BF: brisket fat; SPI: soy protein isolate; CS: cornstarch; MC: moisture content; PC: protein content; FC: fat content; AC: ash content; CC: carbohydrate content; DF: degrees of freedom; A: brisket fat; B: soy protein isolate; C: cornstarch. ^*∗*^*p* < 0.05; ^*∗∗*^*p* < 0.001.
